# The AIDA and the extra laser systems for excimer laser trabeculotomy proved comparable IOP lowering efficacy—12-month results

**DOI:** 10.1007/s10792-021-02140-1

**Published:** 2022-02-04

**Authors:** Sufian Hommayda, Timothy Hamann, Marc Töteberg-Harms

**Affiliations:** 1 Medical Faculty, University of Zurich, Zurich, Switzerland; 2 Department of Ophthalmology, University Hospital Zurich, Frauenklinikstrasse 24, 8091 Zurich, Switzerland; 3 Medical College of Georgia, Augusta University, Augusta, GA USA

**Keywords:** Glaucoma, Minimally invasive glaucoma surgery, Laser, Excimer laser trabeculotomy, Excimer laser trabeculostomy, Cataract, Phacoemulsification

## Abstract

**Purpose:**

Cataract surgery combined with excimer laser trabeculotomy (phaco-ELT) leads to a significant reduction in intraocular pressure by enhancing trabecular outflow. The aim of this study is to compare two laser systems for ELT (AIDA vs. ExTra).

**Methods:**

In this retrospective chart review, inclusion criteria were a diagnosis of glaucoma and phaco-ELT between 07/17/2010 and 07/17/2018. Data were collected preoperatively and postoperatively up to 1 year. Success was defined as IOP reduction of ≥ 20% compared to baseline plus an IOP of < 21 mmHg with no hypotony, no loss of light perception vision, and no subsequent glaucoma surgery.

**Results:**

Three hundred and fourteen eyes (mean age 75.9 ± 8.6 years) were included. Baseline intraocular pressure (IOP) for the ExTra group (94 eyes) was 20.3 ± 5.9 mmHg on 2.0 ± 1.3 anti-glaucoma drugs (AGD) and a best-corrected visual acuity (BCVA, logMar) of 0.5 ± 0.4. For The AIDA group (220 eyes), baseline IOP was 18.7 ± 6.3 mmHg on 2.0 ± 1.3 AGD and a BCVA of 0.3 ± 0.3. In the ExTra group, IOP was reduced to 12.8 ± 2.5 mmHg (−37%) and in the AIDA group to 14.7 ± 3.9 (−21%, *p* = 0.14) at 1 year. AGD reduction in the ExTra group was 1.3 ± 1.5 and 1.8 ± 1.4 in the AIDA group (*p* = 0.14). Success rates were 80% (ExTra) and 70% (AIDA), respectively (*p* = 0.552). Thirty-one eyes (10.0%) required a subsequent glaucoma surgery during the follow-up.

**Conclusion:**

Both laser platforms, the ExTra and the AIDA laser, used for Phaco-ELT lead to a significant reduction in intraocular pressure and anti-glaucoma drugs with no statistically significant difference in success rates during the follow-up of 12 months.

**Trial Registration Swissethics: 2018–01,791.:**

## Introduction

An estimated 2.9 million people are blind due to glaucoma with increasing numbers, making glaucoma one of the leading causes of irreversible blindness worldwide [1]. Between 1990 and 2015, about 14% of all cases of blindness in patients aged 50 years and older in central Europe were caused by glaucoma [2]. Usually, increased resistance in the trabecular meshwork is the reason for decreased aqueous humor outflow and, thus, increased intraocular pressure [3]. The only evidence-based treatment for glaucoma is lowering intraocular pressure to slow down disease progression [4–8]. Reduction in IOP can be achieved by topical and/or systemic medications, laser trabeculoplasty, and various surgical procedures, such as minimally invasive glaucoma surgery (MIGS) procedures and filtering surgeries (i.e., trabeculectomy and glaucoma drainage devices) [9].

MIGS procedures cover a field of evidence-based techniques to treat glaucoma with a lower risk profile compared to trabeculectomy, faster recovery, and, therefore, the reduced necessity of frequent postoperative visits and fewer postoperative complications [10]. MIGS procedures can easily be combined with cataract surgery [11]. Excimer laser trabeculotomy (ELT, or sometimes referred to as excimer laser trabeculostomy) is one of these MIGS procedures, first clinically used by Vogel et al. in 1997 [12–23]. In ELT, a pulsed xenon chloride excimer laser connected to a quartz fiber optic probe non-thermally creates ten laser channels with a diameter of approximately 200–210 μm each through the trabecular meshwork and the inner wall of Schlemm’s canal. Thus, the physiological trabecular (conventional) outflow is increased or restored, and IOP is consecutively reduced. Blood reflux into the anterior chamber at the end of an ELT procedure, which is commonly observed, proves laser channels from the anterior chamber into Schlemm’s canal are patent [11].

The AIDA laser system (TUI-Laser AG, Germering, Germany) was the first commercially available and CE (Conformité Européenne) certified system to perform ELT. Later, in February 2014, the ExTra laser system (MLase AG, Germering, Germany) had been introduced (generation 2) [24]. Both laser systems use a xenon chloride excimer laser with similar laser settings (see Table 1). The AIDA laser system was developed by TUI-Laser AG in the late 90ies and is no longer available. The ExTra laser system was introduced to the market early in 2014 from MLase AG. There is no relationship between MLase AG and TUI-Laser AG except from the fact that some employees have been working for TUI-Laser AG prior to working for MLase AG.

**Table 1:** Technical data of the ELT laser systems

	AIDA	ExTra
Laser type	XeCL excimer laser, laser class 4	XeCL excimer laser, laser class 4
Wavelength	308 nm	308 nm
Cooling	Air	Air
Pulse energy	1.2 mJ at fiber tip	1.3 mJ at fiber tip
Pulse duration	60–120 ns	60–120 ns
Repetition rate	20 Hz	20 Hz
Laser spot size	200 μm	210 μm
Cannula diameter	500 μm	500 μm
Cannula material	Stainless steel	Stainless steel
Length of fiber	200 cm	200 cm

(AIDA = AIDA laser system; ExTra = ExTra laser system; XeCl = Xenonchloride; nm = nanometer(s); mJ = millijoule(s), ns = nanosecond(s); Hz = hertz; μm = micrometer(s); cm = centimeter(s))

The following findings on cataract surgery combined with excimer laser trabeculotomy (phaco-ELT) have been published thus far: (1) ELT and phaco-ELT effectively lowers IOP and reduces the need for topical anti-glaucoma drugs (AGD), (2) ELT is more effective when combined with cataract surgery, (3) phaco-ELT lowers IOP more effectively in higher baseline IOP, however, (4) even when baseline IOP is ≤ 21 mmHg phaco-ELT still lowers IOP, and (5) phaco-ELT can lower IOP for a long period of time, i.e., up to 4 years [13–18, 20–23]. All of these findings are based on studies which have used the AIDA laser system. Only one study which used the ExTra laser system has been published so far [19]. There are no comparative studies of both laser systems to show that findings from the AIDA laser system are transferable to the ExTra laser system. Hence, the aim of this study is to prove similar efficacy of both laser systems.

## Methods

ELT was performed with the AIDA laser system from 07/17/2010 to 07/16/2014 by two surgeons (JF and MTH). From 07/17/2014 onwards, the ExTra laser system was used by two surgeons (JF and MTH). Outcome parameters were defined as IOP, best-corrected visual acuity (BCVA), number of AGD, and the occurrence of complications. Data were collected at baseline and postoperatively at 1 day, 1 week, 1 month, 3 months, 6 months, 9 months, and 12 months.

### Definition of success

Treatment success was defined as an IOP < 21 mmHg and an IOP reduction of ≥ 20% compared to baseline without hypotony (i.e., IOP < 5 mmHg), loss of light perception vision, or subsequent surgery during the follow-up period of 12 months to further reduce IOP. As primary outcome, success rates of ELT treatment with both laser systems were compared. As secondary endpoints, reduction in IOP and AGD was computed.

### Surgical procedure

At the end of a standard clear-cornea phacoemulsification cataract surgery with intraocular lens implantation in the bag, a medical miosis was induced by intracameral injection of 1% acetylcholine chloride (Miochol E, Bausch & Lomb Swiss AG, Zug, Switzerland). Then, a cohesive ophthalmic viscosurgical device (Healon GV, Johnson & Johnson, New Brunswick, NJ, USA) was used to fill the anterior chamber and deepen the angle. Subsequently, the fiber-optic probe of the ELT Laser was coupled to an endoscopic optic and inserted through the 2.4 mm phaco main-incision. The fiber was brought in contact to the trabecular meshwork opposite to the clear cornea incision. The position of the laser fiber tip was controlled via the endoscopic optics. Then, ten laser channels with a diameter of approximately 200 (AIDA)/210 μm (ExTra) each were created (see Table 1 for laser settings) in the inferonasal quadrant. The procedure has been previously described in detail [13, 14, 18, 19, 23].

The difference in distal energy density between AIDA and ExTra is due to the different diameter of the fiber tip (200 vs. 210 μm, see Table 1). To achieve a specific and, thus, comparable treatment efficiency 1.2 mJ at the end of the fiber are required for a fiber diameter of 200 μm (AIDA) and 1.3 mJ for a fiber diameter of 210 μm (ExTra). The energy density is identical, which makes the two laser systems equivalent in regard to output energy density. The pulse length of the XeCl excimer laser is determined by the mechanical and electrical design of the circuit, the laser chamber, and the gas mixture. In fact, both systems have the identical dependency between high voltage and pulse length. 60–120 ns is the right range for both systems.

### Postoperative treatment

A fixed combination ointment of tobramycin (3 mg/ml) and dexamethasone (1 mg/ml) (Novartis, Basel, Switzerland) was applied to the operated eye at the end of the procedure. The eye was then patched and shielded overnight. Starting the next day, tobramycin (3 mg/ml) and dexamethasone (1 mg/ml) fixed combination eye drops (Novartis, Basel, Switzerland) were prescribed q.i.d. and tapered out weekly over 4 weeks. AGD were continued after the procedure and, at the discretion of the surgeons, discontinued during the follow-up period according to the IOP.

### Statistical analysis

Data were collated into a database (Excel 2016, Microsoft, Redmond, WA, USA) and transferred to the statistics program for final data analysis (SPSS IBM, Chicago, IL, USA Version 26.0.0). Descriptive statistics such as mean and standard deviation for continuous variables and absolute and relative frequencies for discrete variables were computed. Differences of IOP and AGD between baseline and 1 year were computed. An independent sample *t *test was used to compare success rates of the two laser systems. The results of statistical analysis with *p* values smaller than 0.05 were considered statistically significant.

## Results

Two patients were excluded due to loss of follow-up or no-available postoperative data. In total, 314 eyes were included in this study with a mean age of 75.9 ± 8.6 (range 40.2–96.4) years. Table 2 provides the distribution and demographical data between the ExTra and AIDA laser. There was no difference regarding gender, eye, glaucoma diagnosis, and previous glaucoma surgery. Ninety-four eyes (29.9%) received laser treatment with the ExTra laser and 220 (70.1%) with the AIDA laser.

**Table 2:** Demographical data of the ExTra laser system and the AIDA laser system group

	ExTra (*n* = 94)	AIDA (*n* = 220)	Total (*n* = 314)	Percentage	*p*
*Gender*					0.195
Male	27 (28.7%)	81 (36.8%)	108	34.4	
Female	67 (71.3%)	139 (63.2%)	206	65.6	
*Eye*					0.805
RE	52 (55.3%)	117 (53.2%)	169	53.8	
LE	42 (44.7%)	103 (46.8%)	145	46.2	
*Procedure*					0.095
ELT	1 (1.1%)	0	1	0.3	
Phako-ELT	93 (98.9%)	220 (100%)	313	99.7	
*Glaucoma diagnosis*					0.425
Primary open angle glaucoma	43 (45.7%)	127 (57.7%)	170	54.1	
Pseudoexfoliative glaucoma	42 (44.7%)	74 (33.6%)	116	36.9	
Pigmentdispersion glaucoma	3 (3.2%)	4 (1.8%)	7	2.2	
Angle closure glaucoma	4 (4.3%)	7 (3.2%)	11	3.5	
Low tension glaucoma	1 (1.1%)	4 (1.8%)	5	1.6	
Other glaucomas	1 (1.1%)	2 (0.9%)	3	1	
*Previous Glaucoma Intervention*	14 (14.9%)	21 (9.5%)	35	11.2	0.177
Visual acuity affecting diagnosis^*^	11 (11.7%)	27 (12.3%)	38	12.2	1.0

(*cataract excluded; AIDA laser system; ExTra = ExTra laser system; LE = left eye; RE = right eye; ELT = excimer laser trabeculotomy; phaco-ELT = phacoemulsification cataract extraction with intraocular lens implantation combined with ELT)

### Intraocular pressure, anti-glaucoma drugs, and visual acuity

There was no difference regarding IOP (*P* = 0.43) and AGD (*P* = 0.98) at baseline. IOP and AGD were lowered throughout the entire follow-up of 12 months with no statistical difference between the two laser platforms (see Table 3). IOP was lowered by −37% in the ExTra and by −21.0% in the AIDA group at 12 months. There was no significant difference in BCVA at twelve months between both groups (0.3 ± 0.4 in the ExTra group and 0.3 ± 0.3 in the AIDA group; *p* = 0.87). We also compared the two largest subgroups, primary open-angle glaucoma and pseudoexfoliative glaucoma. There is no difference in regard to IOP, AGD, and BCVA at any time point with the exception of IOP at 1 week, which was significantly lower in the pseudoexfoliative glaucoma subgroup (13.9 ± 5.8 vs. 17.0 ± 6.3, *p* = 0.025). However, the difference was not present at the later time points.

**Table 3:** Results

	Baseline Mean ± SD	*p* (ExTra vs. AIDA)	1 day	*p* (compared to baseline)	*p* (ExTra vs. AIDA)	1 week	*p* (compared to baseline)	*p* (ExTra vs. AIDA)	1 month	*p* (compared to baseline)	*p* (ExTra vs. AIDA)	3 month
IOP [mmHg]												
ExTra	20.3 ± 5.9		17.8 ± 8.4	0.02		15.4 ± 6.0	< 0.001		14.9 ± 4.9	< 0.001		13.7 ± 4.4
AIDA	18.7 ± 6.3		18.5 ± 8.5	0.44		15.9 ± 7.2	0.02		16.3 ± 5.3	< 0.001		15.8 ± 4.7
		0.43			0.53			0.7			0.15	
AGD												
ExTra	2.0 ± 1.3		1.5 ± 1.4	< 0.01		1.6 ± 1.6	0.09		1.6 ± 1.3	0.19		1.5 ± 1.5
AIDA	2.0 ± 1.3		1.3 ± 1.4	< 0.001		1.3 ± 1.5	0.002		1.6 ± 1.5	0.07		2.1 ± 1.5
		0.98			0.28			0.43			0.92	
BCVA [logMAR]												
ExTra	0.5 ± 0.4		0.6 ± 0.3	0.01		0.4 ± 0.3	0.05		0.3 ± 0.3	0.003		0.2 ± 0.2
AIDA	0.3 ± 0.3		0.4 ± 0.3	< 0.001		0.4 ± 0.3	0.74		0.3 ± 0.3	0.03		0.3 ± 0.3
		0.004			0			0.65			0.83	

	*p* (compared to baseline)	*p* (ExTra vs. AIDA)	6 month	*p* (compared to baseline)	*p* (ExTra vs. AIDA)	9 month	*p* (compared to baseline)	*p* (ExTra vs. AIDA)	12 month	*p* (compared to baseline)	*p* (ExTra vs. AIDA)
IOP [mmHg]											
ExTra	< 0.001		14.3 ± 2.7	< 0.001		12.8 ± 2.8	< 0.001		12.8 ± 2.5	0.002	
AIDA	< 0.001		15.0 ± 4.3	< 0.001		14.7 ± 2.5	0.02		14.7 ± 3.9	0.002	
		0.06			0.51			0.04			0.14
AGD											
ExTra	0.11		1.5 ± 1.5	0.04		2.0 ± 1.5	0.07		1.3 ± 1.5	0.04	
AIDA	0.74		2.2 ± 1.4	0.08		1.8 ± 1.4	0.03		1.8 ± 1.4	0.01	
		0.89			0.84			0.65			0.3
BCVA [logMAR]											
ExTra	0.001		0.3 ± 0.4	0.02		0.2 ± 0.2	0.01		0.3 ± 0.4	0.15	
AIDA	0.17		0.2 ± 0.2	0.003		0.2 ± 0.3	0.54		0.3 ± 0.3	0.03	
		0.14			0.33			0.57			0.87

(AIDA laser system; ExTra = ExTra laser system; IOP = intraocular pressure; AGD = anti-glaucoma drugs; BCVA = best-corrected visual acuity; mmHg = millimeters of mercury)

### IOP spikes

IOP spikes (i.e., an increase of IOP equal to or greater than 10 mmHg from baseline) were found in 41/314 cases (13.4%) at the 1 day postop visit, in 3/314 cases (1%) at the 1 week postop visit (see Table 4), and in 3/314 cases (1.6% at the 1 months visit. Over the whole first months, IOP spikes occurred in 44/314 eyes during the first postoperative month (14.0%). There was no statistical significant difference in the occurrence of IOP spikes between the AIDA and the ExTra laser system (see Table 4).

**Table 4:** IOP spikes

	ALL (*n* = 314)	EXTRA (*n* = 94)	AIDA (*n* = 220)	*P*
1 day	41 (13.4%)	10 (10.6%)	31 (14.1%)	0.407
1 week	3 (1.0%)	1 (1.1%)	2 (0.9%)	0.898
1 month	4 (1.6%)	1 (1.1%)	3 (1.4%)	0.829
1 d to 1 month	44 (14.0%)	11 (11.7%)	33 (15.0%)	0.442

(AIDA = AIDA laser system; ExTra = ExTra laser system; IOP = intraocular pressure; IOP spike = increase of IOP ≥ 10 mmHg from baseline)

### Complications

A cystoid macular edema occurred in 5/314 eyes (2%) after ELT and a hyphema > 1.0 mm in 5/314 cases (2%). 31/314 eyes (10%) required subsequent surgery to lower IOP during the entire follow-up of 12 months. Out of these 31 eyes, 14 underwent trabeculectomy, 15 cyclophotocoagulation, one had an iStent Inject implantation, and one a CyPass implantation. In addition, 6 eyes had selective laser trabeculoplasty after ELT during the follow-up to further lower IOP. All 6/314 (2%) cases of ocular hypotony resolved spontaneously up to the 1-month visit, with 2 such cases having a choroidal effusion, which in one case led to a suprachoroidal hemorrhage during the first night after surgery and the need for a pars plana-vitrectomy with drainage of the hemorrhage. The two dislocated IOLs (iris capture) in the study population were repositioned and two eyes with vitreous loss and a vitreous strand trapped in a paracentesis were successfully treated by Nd:YAG laser.

### Success

At 12 months, the success rate in the ExTra laser system group was 80% and in the AIDA laser system group 70%. The difference in success rates between the groups was not significant (*p* = 0.552).

## Discussion

The study confirmed the IOP lowering efficacy of combined phaco-ELT treatment in both laser groups after a follow-up of 12 months postoperatively (ExTra − 37%, AIDA − 21%) with no significant difference (*p* = 0.14). Average number of required AGD was reduced from 2.0 ± 1.3 to 1.5 ± 1.5 in the ExTra group and to 1.8 ± 1.4 in the AIDA group. The low number of required subsequent glaucoma surgery to further lower IOP (10%) proves that phaco-ELT is a considerable therapy to lower IOP and AGD, with high safety profile during the 1st year of follow-up.

Babighian et al. [16] proved in a single center randomized controlled trial over a 2 year follow-up the IOP lowering effect of ELT (*n* = 15) versus SLT (*n* = 15) in mild to moderate POAG. Complete success was defined as a ≥ 20% reduction in IOP without glaucoma medications (meds) and qualified success as ≥ 20% reduction in IOP with meds. Compared to other studies, only 8 ELT laser spots were applied. In the ELT group, complete success was achieved in 53%, qualified success in 87%. Baseline mean IOP was decreased from 25.0 ± 1.9 mmHg to 17.6 ± 2.2 mmHg (30% reduction, *p* < 0.0001) and a reduction in medication 2.3 ± 0.7 to 0.7 ± 0.8 (*p* = 0.005).

Töteberg-Harms et al. performed a 1-year prospective case series on 64 consecutive eyes on patients with open angle glaucoma undergoing combined phaco-ELT [14]. They divided the study population into two groups based on preoperative IOP with group 1 having IOP ≤ 21 mmHg and group 2 having IOP > 21 mmHg. IOP dropped after the treatment from 19.8 ± 5.3 mmHg to 15.2 ± 4.4 mmHg (23% reduction): group 1 IOP from 16.5 ± 2.9 mmHg to 14.6 ± 3.7 mmHg (12% reduction) and group 2 from 25.8 ± 2.9 mmHg to 16.4 ± 5.4 mmHg (37% reduction). Compared to baseline, use of AGD was reduced after one year (group 1: 2.5 ± 1.0 to 1.4 ± 1.3 group 2: 2.2 ± 1.4 to 1.6 ± 1.5). In 2011 Töteberg-Harms et al. also reported about the IOP lowering effect of ELT combined with cataract surgery with a follow-up time of 12 months, (IOP 25.33 ± 2.85 mmHg to 16.54 ± 4.95 mmHg, 34.7%) AGD were reduced from 2.25 ± 1.26 to 1.46 ± 1.38 [18]. Again Töteberg-Harms et al. published in 2017 a 4-year retrospective interventional case series comparing ELT + CE and trabeculectomy + CE in patients with OAG [13]. In the ELT + CE group (*n* = 51) IOP was at baseline 19.0 ± 9.0 mmHg (median ± interquartile range, IQR) on 2 ± 1 meds and decreased to 15.0 ± 5.0 mmHg on 1 ± 2 meds at 1 year and further to 14.0 ± 5.5 mmHg on 1 ± 2 meds after 4 years. Complete success was defined in this study as IOP ≤ 21 mmHg and ≥ 20% IOP reduction without meds and qualified success as IOP ≤ 21 mmHg and ≥ 20% reduction in IOP with meds. In the ELT + CE group, complete success rate was at 1 year 18% and at 4 years 9%. Qualified success was at 1 year 47% and at 4 years 34%.

Jozic et al. compared in a 1-year retrospective interventional case series CE with ELT + CE and Trabectome + CE in patients with OAG [19]. They compared CE vs ELT + CE versus Trabectome + CE. ELT + CE group showed a better reduction in medication (1.4 ± 0.7 meds to 0.5 ± 0.8, *n* = 105) and improved mean survival time (Kaplan–Meier curve, (20.6 ± 1.0 months) compared to the other groups. IOP dropped from 17.8 ± 4.3 to 13.2 ± 2.3 mmHg (34.8%). Deubel et al. showed in a recent study [25] an IOP lowering effect of almost 30% after 1 year (25.50 mmHg to < 18.0 mmHg).

Our results confirm the outcome of previous studies [13, 14, 18] and prove a significant reduction in IOP and AGD comparable with the studies mentioned above. The higher IOP reduction in the ExTra group compared to the AIDA group can be explained by the higher baseline IOP in the ExTra group; the IOP lowering potency of ELT depending on baseline IOP was previously described by Töteberg et al. [14].

The primary endpoint might be influenced by comorbidity, already performed glaucoma-surgery, age, sex, varying types of glaucoma, lens status (cataract, phacic, aphacic, pseudophacic) or combination of the above. The secondary endpoints of the study are reduction in IOP and anti-glaucoma drugs by ELT. Therefore, post-interventional IOP and history of medication will be evaluated in this regard. Since topical or systemic glaucoma medications have side effects and are difficult to apply for many patients, especially those with impaired visual or coordinative ability, a reduction in antiglaucomatous therapy is another aim of MIGS and, therefore, besides IOP the secondary endpoint of this proposed study. The secondary endpoint might be influenced by compliance, medication, comorbidity, already performed glaucoma-surgery, age, sex, various types of glaucoma, lens status (cataract, phakic, aphakic, pseudophakic) or by a combination of the above.

The appearance of postoperative IOP spikes especially at the first day after surgery (see Table 4) could be explained by remaining viscosurgical device and a microhyphema. This can be prevented by a thorough irrigation/aspiration at the end of the procedure. In addition, intravenous or oral acetazolamide can be administered at the beginning, during, or immediately after the procedure.

The limitations of this study are its retrospective design, the short follow-up of 12 months. In addition, the number of eyes treated is unevenly distributed between the Extra and the AIDA laser with more procedures in the ExTra laser group. Because of the retrospective study design, IOP measurements were performed only once at each time point and not averages out of three measurements of a diurnal IOP curve as it is usually done in randomized controlled glaucoma trials.

## Conclusion

Our study has proven in a large population no significant difference in outcomes between the AIDA and the ExTra laser system to perform ELT during the follow-up of 12 months. Thus, the findings from previous publications with the AIDA laser platform seem to be transferable to the ExTra laser platform. Both laser systems showed a favorable safety profile of this implant-free MIGS procedure. Furthermore, ELT can be offered as a therapy for phacic patients who are scheduled for cataract surgery to reduce IOP and reduce the need for topical medications. Previous studies proved an IOP lowering efficacy of combined phaco-ELT which exceeds the efficacy of phaco alone [13–23, 26].

## Data Availability

The data that support the findings of this study are available from the corresponding author upon reasonable request and within national and international laws.
